# Sleeping in Church

**Published:** 1876-01

**Authors:** 


### SLEEPING IN CHURCH.

MOST persons know the distresssing effort it requires, sometimes, to keep awake, during the delivery of a long and not particularly interesting sermon, on a summer's day; various expedients have been devised to remedy the indecency, such as pinching one's self, holding one foot three or four inches above the floor, or taking a short nap immediately before going to church. One of the most quiet, efficient, and prompt remedies we have yet fallen upon, is to put into the vest-pocket, a teaspoonful of the powder of pure cayenne pepper, and when any drowsiness is experienced, put a good pinch of it in the mouth. Some enterprising apothecary might make a hit by fabricating Red Pepper Lozenges.

